# Genetic testing for monogenic forms of motor neuron disease/amyotrophic lateral sclerosis in unaffected family members

**DOI:** 10.1038/s41431-024-01718-4

**Published:** 2024-11-05

**Authors:** Jade Howard, Amina Chaouch, Andrew G. L. Douglas, Rhona MacLeod, Jennifer Roggenbuck, Alisdair McNeill

**Affiliations:** 1 Division of Neuroscience and Neuroscience Institute, The University of Sheffield, Sheffield, UK; 2 Manchester Centre for Clinical Neuroscience, Salford Royal NHS Foundation Trust, Northern Care Alliance, Stott Lane, M6 8HD UK; 3 Oxford Centre for Genomic Medicine, Oxford University Hospitals NHS Foundation Trust, Oxford, UK; 4 Nuffield Department of Clinical Neurosciences, University of Oxford, Oxford, UK; 5 Division of Evolution, Infection and Genomics, School of Biological Sciences, The University of Manchester, Manchester, UK; 6 Manchester Centre for Genomic Medicine, Manchester University NHS Foundation Trust, Manchester, UK; 7 Department of Neurology, The Ohio State University Wexner Medical Center, Columbus, USA; 8 Sheffield Clinical Genetics Service, Sheffield Children’s Hospital NHS Foundation Trust, Sheffield, UK

## Abstract

Motor neuron disease (MND), also referred to as amyotrophic lateral sclerosis (ALS), is a monogenic disease in a minority of cases, with autosomal dominant inheritance. Increasing numbers of people with MND are requesting genetic testing, and indeed receiving a genetic diagnosis. Consequently, requests for genetic counselling and predictive testing (i.e. of unaffected family members) are similarly expected to rise, alongside pre-symptomatic clinical trials. Despite this, there is no evidence-based guideline for predictive genetic testing in MND. This paper provides an overview of the genomic basis of MND, focusing specifically on the most common monogenic causes of MND. It then lays out the complexities of MND predictive testing, including the genetic landscape characterised by incomplete penetrance, clinical and genetic heterogeneity, and an oligogenic mechanism of pathogenesis in some cases. Additionally, there is limited research on the psychosocial impact of predictive genetic testing for MND, with studies suggesting potential difficulty in adjusting to the news, in part due to a lack of support and follow-up. This underscores a case for evidence-based, disease-specific guidance for predictive testing in MND.

## Introduction

Motor neuron disease (MND), also referred to as amyotrophic lateral sclerosis (ALS), is a relentless neurodegenerative disease that primarily affects motor neurons. It leads to progressive muscle weakness and death from respiratory failure within an average of 2–5 years of symptom onset. MND is relatively rare, with an incidence of 2 per 100,000 person years. There is emerging evidence that the incidence of MND is increasing with some predicting ~400,000 people will live with MND globally by 2040 [1].

Genomic science has contributed greatly to our understanding of MND. Although around 85–90% of MND cases are seemingly sporadic, MND is a monogenic disease in around 10-15% of cases. Since the identification of SOD1 through linkage and candidate gene sequencing, multiple single gene causes of MND have been identified using increasingly sophisticated testing platforms [2]. We provide an overview of monogenic causes of MND, with a focus on issues relevant to predictive testing.

## Clinical presentation and diagnosis of MND

People with MND typically report weakness which can start in one limb and progress to other contiguous limbs. People can also present with speech and swallowing problems, and sometimes early respiratory failure. Some individuals may exhibit emotional lability with change in personality and behaviour alongside a reduction in verbal fluency (frontotemporal dementia [FTD] spectrum). The average time before diagnosis from the onset of symptoms has remained at around one year over the past 30 years [3].

MND remains a clinical diagnosis, and neurological examination typically shows a combination of upper motor neuron (UMN) and lower motor neuron (LMN) signs. Imaging of the brain and spinal cord is often normal and neurophysiological studies can show features of muscle denervation, often combined with evidence of chronic reinnervation. The latter are not specific to MND and can be found in other LMN syndromes. There are currently no specific diagnostic tools for MND. Most neurologists will investigate patients to exclude potential mimics, which may be treatable. These include inflammatory motor neuropathies, inclusion body myositis, cervical myeloradiculopathy, spinal and bulbar muscular atrophy (Kennedy disease) and spinal muscular atrophy (SMA) [4–6].

To be able to provide support and well-coordinated care, MND clinics have to work in close partnership with several health and social care community teams and local long-term ventilation services. In the UK, the National Institute for Health and Care Excellence (NICE) has set specific guidance (N42) tailored to address the complex care needs of people living with MND, including nutrition and gastrostomy feeding, ventilation, communication aids, physical therapy, cognition, psychology (families/carers) and palliative care [7]. People living with MND are usually reviewed in neurology clinics every three months and have access to different healthcare professionals through the course of their disease.

MND remains incurable with only one licensed treatment in the UK, riluzole, extending life by 3–4 months [8]. Since its introduction in 1994, multiple clinical trials for other potential treatments have failed to lead to drug approval. Edaravone, a treatment for acute stroke, was approved in several countries since a clinical trial published in 2017 suggested it slowed progression of symptoms in the early stage of the disease [9, 10]. The first genetically targeted treatment, tofersen, for the treatment of *SOD1* MND has been approved in the USA and elsewhere [11], with a decision pending in the UK. Phase 1–3 trials of other antisense oligonucleotide treatments targeting common MND-linked gene variants are ongoing [12].

## The genomic basis of MND

MND displays considerable clinical and genomic heterogeneity. Pathogenic variants in over 40 genes have been associated with development of MND, most of which are inherited in an autosomal dominant pattern, with reduced penetrance. This accounts for around 70% of familial MND, with around 30% of cases unexplained, in spite of a presumed monogenic form of the disease based on family history [13]. The clinical spectrum in MND-linked variants incorporates a range of motor phenotypes, and in some cases, frontotemporal dementia (FTD) and other neurological and psychiatric symptoms. Variants in *C9orf72*, *SOD1*, *FUS*, and *TARDBP* are the most prevalent. However, the genomic architecture of MND varies with geography: the *C9orf72* repeat expansion affects more individuals of European ancestry, whilst *SOD1* variants are more common in people of Asian ancestry [14]. The causal role of many of MND-linked pathogenic variants remains uncertain or disputed.

MND is sometimes classed as either familial or sporadic. Sporadic MND is defined as a singleton case within the family, with no other affected relatives. However, there is no clear definition of familial MND, with a lack of consensus across the research and clinical community [15]. There is no agreed number of affected individuals with MND to define familial disease, nor is there agreement on whether individuals with other clinical presentations of neurodegeneration should be classed as affected. Further, pathogenic genetic variants are identified in people with MND who have a clear, dominant family history of the disease, and people with MND who have no known family history, though there is a higher prevalence of pathogenic gene variants in the former group [2, 15].

There are several factors which can explain the absence of a family history in individuals with MND linked to a monogenic aetiology. Phenotypic heterogeneity can obscure a family history, for example, a diagnosis of FTD in a relative may not be classified as significant or indicative of a family history of MND. Many genetic forms of MND show age-related penetrance, and it is possible that people can die from unrelated causes before manifesting MND. Estrangement, secrecy and non-paternity within families can also hide a family history of MND [16]. De novo variants in the person with MND would mean that there would be no history of the disease in preceding generations. These variants can be identical to missense variants reported in familial MND [17]. As such, it has been questioned whether the distinction between supposedly sporadic and familial disease is outdated [15].

Given the large number of potential causal genes, genomic diagnosis of MND requires genome or exome based testing (rather than single gene testing in Huntington’s Disease). Such testing has only relatively recently been implemented in most healthcare systems. In many settings, neurology clinicians are undergoing a period of upskilling to permit routine deployment of genome sequencing in MND.

Genome-wide association studies (GWAS) have identified multiple genomic loci associated with increased risk of MND, some of which overlap with known monogenic causes [18]. A polygenic risk score for MND has been constructed from GWAS data, but it only explains a fraction of MND heritability in the studied population [19]. Predictive genetic testing refers to testing for causal monogenic variants and not loci from GWAS or polygenic risk scores. This is because the predictive validity of polygenic risk scores is currently too low to be clinically relevant.

### SOD1

Through successful linkage analysis, *SOD1* was identified as the first monogenic cause of MND in 1993 [20]. *SOD1* variants account for 1–2% of sporadic cases and 10–15% of familial cases in people from a white European background. More than 200 pathogenic variants have been identified, most of which lead to inheritance of the condition in an autosomal dominant fashion.

*SOD1* exhibits great phenotypic heterogeneity but certain variants have been linked to a more severe dominant phenotype, such as p.Gly73Ser and p.Ser106Leu. On the other hand homozygous variants (p.Asp91Ala) in *SOD1* have been described in more slowly progressive MND cases with some atypical features including sensory and bladder symptoms.

*SOD1* encodes superoxide dismutase 1, a cytosolic Cu/Zn binding protein. Pathogenic variants in this gene were shown to cause a toxic gain of function with different pathways being involved including oxidative stress, mitochondrial dysfunction, excitotoxicity and axonal transport disruption [2]. Recently, an antisense oligonucleotide (tofersen) targeting the *SOD1* mRNA was shown to lower protein synthesis and cerebrospinal fluid (CSF) neurofilament levels with some clinical improvement noted after 6 months [12, 21]. This treatment received accelerated approval by the FDA [11]. NICE approval is still pending in the UK.

### C9orf72

In 2011, the *C9orf72* repeat expansion was identified, through linkage studies, in several families with MND and FTD [22, 23]. This gene has since been confirmed to be the most common genetic cause of MND and FTD in individuals of European ancestry, accounting for up to 40% of familial and 7% of sporadic MND. The *C9orf72* expansion of a hexanucleotide GGGGCC repeat encoding dipeptide repeats exhibits variable phenotypic expressivity; individuals can develop MND and FTD (or both), and there are also associations between *C9orf72* and parkinsonism, psychosis, and Huntington’s disease (HD) like phenotypes [24, 25].

Studies have suggested that most *C9orf72* MND patients carry more than 700 to 1600 repeats in their pathogenic allele [22]. However there does not seem to be a correlation between the size of the repeat expansion and the clinical phenotype or age of onset as seen in other trinucleotide repeat diseases like HD. The underlying pathomechanism underpinning *C9orf72* MND remains elusive but probably involves a combination of toxic gain of function disturbing RNA metabolism, loss of function and dipeptide repeat protein (DPR) accumulation [2]. Research studies focused on targeting the *C9orf72* repeat expansion using antisense oligonucleotides have revealed clear reduction in transcripts in a transgenic mouse model, with reduction in protein biomarkers in patient CSF. However, clinical trials in patients have so far failed to show any functional improvement between the treatment and placebo groups [12].

## Other rare monogenic causes of MND

Pathogenic repeat expansions in genes associated with other neurodegenerative diseases have also been linked with MND. In an Australian study, repeat expansions in 20 neurodegeneration linked genes were genotyped in MND patients. In 17.6% there was a pathogenic repeat expansion in a neurodegenerative disease gene (excluding *C9orf72*). However, after statistical correction, only the *ATXN2* repeat expansion (intermediate range) was significantly enriched in MND. Other studies have suggested that pathogenic repeat expansions in *HTT* may be associated with an MND/MND-FTD presentation. Dewan et al. [26] identified 3 cases of MND with pathogenic *HTT* expansions, equating to 0.12% of the studied MND cohort. In a neuropathological study, 0.8% of HD brains had co-existing evidence of MND neuropathology [27]. Repeat expansions in *ATXN2* of intermediate size (29–33 CAG) and in *ATXN1* of full size ( > 33 CAG repeats) have been associated with an increased risk of MND in several population studies [2]. It is not clear if these findings represent phenotypic variability (with a true causal role for these repeat expansions in MND), clinical misdiagnosis or mere coincidence.

There is evidence that a digenic or oligogenic mechanism of pathogenesis operates in some MND families. A recent UK-based study found that 13% of people with MND carried more than one MND-linked variant (pathogenic or VUS) through genome sequencing [28]. In an Australian study of sporadic MND, 6.8% carried plausibly pathogenic variants in two or more MND-linked genes: mostly the *C9orf72* expansion plus a single nucleotide variant in another gene [29]. A Dutch study identified oligogenic variants in around 5% of familial MND [30]. A Chinese study identified that 2.8% of sporadic MND patients had a rare variant in more than one MND-linked gene [31]. Some, but not all, studies support an earlier age of onset of MND for individuals with more than one pathogenic variant. Other studies have found no evidence for an oligogenic mechanism [32].

## Current practice for genomic testing in people affected by MND

International surveys of clinician practices have found that genetic testing is increasingly offered to persons with familial MND, but only 10–50% of those with apparently sporadic MND are offered testing [13, 33–36]. In the UK, everyone diagnosed with MND is eligible for genomic testing, with reporting of a panel of neurodegeneration linked genes on a whole genome sequencing backbone [37]. Surveys of people with MND show that genetic testing is valued and desired, whether or not their MND is familial or sporadic [38, 39]. However, families affected by MND have indicated a need for information on the availability of genetic testing, counselling on the possible implications of test results, and support around family communication. There is also a need for information for their relatives around predictive testing [16, 39, 40].

European guidelines initially recommended that genetic testing for MND should be offered only to patients with familial disease or for the *SOD1* p.(Asp91Ala) phenotype [41]. US care guidelines (reaffirmed February 25, 2023) do not mention genetic testing as a component of MND care [42]. Recently, a US-based expert panel developed evidence‐based, consensus guidelines to provide a framework for the offer of genetic testing to people with MND and to highlight the information that should be provided before and after testing [43]. These recommendations include the offer of single step, comprehensive genetic testing (via multigene panel, exome or genome testing together with an assay to detect the *C9orf72* expansion) for all people with MND. Pre- and post-test risk assessment and genetic counselling considerations are also outlined in detail [43]. Recently, the European Academy of Neurology, in collaboration with the European Reference Network for Neuromuscular Disease, endorsed these guidelines [44]. However, predictive testing for relatives of people with MND is not addressed.

## The development of predictive testing pathways for Huntington’s disease: implications for MND

Predictive testing in HD presents a useful comparison for MND, given both are adult-onset neurodegenerative conditions with limited treatments and no preventative options. Predictive testing for HD was available by linkage in a small number of centres from 1986 [45]. International cooperation through meetings and the formation of a committee involving members of both the International Huntington’s Disease Association (IHA) and the World Federation of Neurology (WFN) Research Group led to the development of recommendations for the process of predictive test counselling from pre-test through to post-test counselling support. The recommendations were updated following the identification of the *HTT* trinucleotide repeat expansion in 1993 [46, 47]. An important aspect of early predictive testing was the systematic evaluation of the impacts of predictive testing carried out within a research framework. The guidelines were further updated in 2013 [48] in response to a review of the research evidence underpinning several sections of the guidance (e.g., intermediates allele results and requests for testing from young people < 18 years). The review was initiated by the EHDN Working Group ‘Genetic Counselling and Testing’ and delivered by a writing committee comprising members of the IHA and WFN research group and EHDN WG.

## Why is predictive testing for MND more complex than for HD?

The genomic and clinical aspects of MND make predictive genetic testing significantly more complex than for HD (Table 1) [16]. Adult-onset HD has a relatively homogenous clinical presentation, with a well-defined natural history of early cognitive and behavioural changes followed by development of the motor disorder (characteristically choreo-athetosis) and autosomal dominant inheritance with high penetrance [49]. Predictive genetic testing for HD is well established in most developed countries’ healthcare systems, and there are active patient support organisations which foster a culture of openness around genetic testing conversations. There is an extensive research literature on predictive testing in HD. Patient preferences, including motivations, and the impact of testing are relatively well studied [50–53]. There is an established international guideline and practice for predictive testing in HD [48]. In comparison with HD, research and support services around MND are less established. The MND community is relatively less familiar with genetic testing options for people with MND and predictive testing options for their relatives, and the benefits, drawbacks and implications of these options.

**Table 1:** Key areas to cover in predictive test counselling.

Pre-Test	Test	Result	Post Test
Information about the condition including inheritance and penetrance Testing options Predictive test protocol Expectations and motivation to test Support system/family communication Rehearsal of possible test outcomes and impacts Potential for insurance discrimination Timing of test Communication of result (e.g., who they plan to share result with) Research opportunities	Consent Plans for result session and follow up	Test outcome & meaning of result for individual and family members (including residual uncertainty) Review of penetrance Plan for follow up Summarise points in a letter or electronic communication	Contact usually within 6–8 weeks of result Assess coping Discuss family communication Options to participate in research

## Special clinical considerations for predictive genetic testing in MND

In the absence of an international protocol for predictive testing in MND, most clinical genetic services adapt the recommendations for predictive testing in HD [16]. However, as highlighted, there are important differences for clinicians to consider in relation to MND. Further, protocols will need to be flexible as and when pre-symptomatic treatment trials become available. The broadly agreed principles for predictive testing for serious neurogenetic conditions, for which there are currently no effective treatments, include genetic counselling as part of the process before, during and after testing, with the blood sample not being taken at the first appointment. Effective predictive testing will be tailored to the individual. The table below includes key areas which should be covered in the course of predictive test counselling.

### Offering predictive testing in families with no identified genomic variant

There is confusion among both MND families and clinicians about who is eligible for predictive testing. Adults who have a family member identified with a pathogenic variant in an MND-linked gene are candidates for predictive testing. It is the authors’ experience that many relatives seek predictive testing even when no causal variant has been identified in their family. Offering a predictive genetic test to a person with a family history, but no identified gene variant, has the potential to be very misleading. Given the genomic heterogeneity of MND, testing an unaffected relative for variants in a panel of MND-linked genes might prove inaccurate if the causal gene in the affected relative was not included. In addition, MND may in the future be conceptualised as an oligogenic condition, given the aforementioned evidence that a minority of people with MND carry two or more potentially causal variants [28]. Without knowing the combination of variants which are pathogenic in a family, it could be difficult to interpret the results of predictive testing. Further, if a variant of uncertain significance (VUS) was identified in the relative with MND, this may add additional uncertainty and stress for the individual. Therefore, testing for VUSs is not recommended unless there is evidence that the variant may in fact be pathogenic. Since around 30% of familial cases of MND remain genomically unsolved, genetic testing can only be used to confirm a genetic cause of MND and not rule out a monogenic aetiology.

### Reduced penetrance of MND-linked genes

Given the known incidences of MND and FTD and the likelihood of finding causative gene variants in both familial and sporadic scenarios, pathogenic and likely pathogenic variants in MND-linked genes are overrepresented in population cohorts and databases such as gnomAD [54]. Maximum population-level penetrance figures for *C9orf72*-related MND/FTD are around 33%, meaning that at least two-thirds of individuals carrying the repeat expansion in the general population will not go on to develop the disease. A similar reduced disease penetrance effect appears to apply to other MND genes, including *SOD1* (54%), *TARDBP* (38%) and *FUS* (19%) [54]. Specific variants, for example *SOD1* p.Ala5Val, are commonly reported to be associated with high-penetrance disease. However, while variant-level effects on disease penetrance are indeed likely to a degree, such reports may be prone to ascertainment bias since historically it has tended to be exactly such high-penetrance families who are likely to have had genetic testing. Reduced penetrance can introduce complications for family members considering predictive testing, and should be carefully explained during genetic counselling to help them weigh up the utility of predictive testing and consider their own tolerance for uncertainty.

### Clinical actionability of genomic variants

For most MND-linked genes, there are no specific disease modifying therapies. However, where there is the possibility of accessing a clinical trial or genetically stratified treatment, care must be taken that this does not unduly bias the consult and’s decision making. Of particular relevance is tofersen for *SOD1* variant carriers. Individual decision making may be biased in favour of having a predictive test, with insufficient attention paid to the negative emotional impact of testing. It should be remembered that presymptomatic treatment of MND remains at the clinical trial stage, and that presymptomatic people are not guaranteed entry into a clinical trial solely based upon their genotype.

### Variable phenotypic expression of MND-linked genes

Giving advice on what clinical features an individual who is found to carry an MND-linked gene variant will develop is challenging. The motor phenotype of MND can vary, even within a family. There is a well characterised association between several monogenic causes of MND and other neurological and psychiatric presentations, but no reliable means of advising a consultand as to which condition they would develop if they have an MND-linked gene variant [55]. Clearly, if, in a given family, MND is associated with a rare genetic variant, then counselling on likely clinical manifestations becomes even more complex.

### Lack of evidence on psychosocial impact of predictive genetic testing for MND

There is extensive evidence on the psychosocial impact of predictive genetic testing for HD. In contrast, the research evidence in MND is limited - making it difficult to appreciate the risk: benefit ratio for predictive testing in these families.

Qualitative studies have given some insight into experiences of MND predictive testing. The period of contemplating the predictive testing decision, waiting for results, and receiving the result are points where thoughts of MND and worries about the future may be particularly challenging and prominent [56].

A positive (abnormal) result may be met with immediate feelings of distress, devastation, and worry and guilt for children, although feelings may become more moderate over time. A 2011 study on MND predictive testing in a US context suggested that emotional upset after receiving results was temporary, with thoughts of suicide one of many transient emotional responses [57]. However, recent research suggests a positive result and lack of support afterward can contribute to serious mental health implications, including suicide attempts. [58]. In the longer term, a positive result can have various ongoing impacts, including a sense of uncertainty, anxiety, dread, hypervigilance over possible symptoms, and anticipatory grief related to future losses. Knowledge of increased risk may present a threat to one’s sense of self and identity, hopes for the future, and ability to fulfil family roles, related to physical and cognitive symptoms that could manifest. Feelings may be grounded perceptions of living with and dying from MND and/ or FTD, at times based on experiences of family members. Impacts on family dynamics and relationships have also been reported, including an inability to maintain relationships, challenges over communication and a sense of isolation, particularly when other relatives are untested or have received a negative (normal) result [56, 59].

However, people may experience both losses and gains on receiving a positive result. Participants have reported that they do not regret their decision, and perceived benefits to having the knowledge and getting an answer. Over time, people were able to use the results to move forwards with their lives and goals [58]. Some described having clearer priorities and perspectives, a changed attitude to life, with a focus on living life to the full, and a renewed motivation to look after their health [56, 57].

Individuals receiving negative (normal) results have also expressed mixed emotions, including gratitude, relief, a sense of opportunity, and guilt and concern over sharing the news with family. Whatever the outcome, it can take varying amounts of time for the individual and family to process the result and adjust [57, 58].

Notably, perceiving oneself as ‘at risk’ can have significant but fluctuating emotional and psychological impact regardless of whether there is an identified genetic variant in the family or whether the individual has undergone predictive testing [56].

### Lack of resources to support genomic testing conversations

In many healthcare settings, offering genome sequencing to people with MND in mainstream neurology clinics is relatively novel, with numerous organisational challenges [60]. In addition, the increased number of genomic diagnoses being made in MND families has resulted in an increased number of referrals to clinical genetics services for predictive testing. Predictive testing for MND is a more recent area which many clinical genetics and neurology services are relatively unfamiliar with. A survey of English neurology clinicians identified low levels of self-reported genomics knowledge and genetic counselling skills. This included poor understanding of the rationale for, and process of, predictive genetic testing for unaffected relatives [60]. This is a potential barrier to at risk relatives accessing predictive testing. In a freedom of information request survey of MND care centres and regional clinical genetics centres, no specific resources were identified to support genomic testing discussions with MND families or information sharing around predictive testing [61].

### Lack of post-test support for those who have an MND-linked gene variant

In a UK context, offers of post-test support for predictive testing may be variable and are often reported to be self-directed. This can lead to feelings of being abandoned or in “a dip”, and can contribute to serious psychological implications, as described above. Where people have been able to access post-test follow-up (e.g., through genetic counsellors), it could be perceived as inadequate for their situation. Uncertainty over how to access support, awareness that others may be receiving more, and the contrast between the support over the genetic counselling process and that available after can all exacerbate such feelings [58].

Surveillance can be reassuring for people who have received a positive result [59]. The option of an annual clinical follow up appointment, regardless of willingness to participate in research, may be a useful development in post-test support. It is vital that there is a clear referral pathway for individuals with MND-linked gene variants who develop potential symptoms.

Generally, the need for improved post-test support has been emphasised, with suggestions including mandatory tailored counselling sessions, peer support opportunities, ongoing monitoring, and an information sheet with “next steps”, including how to participate in research [58]. Pathways of care are in their infancy, and it is hoped that establishing these within a wider multi-disciplinary team, including clinical psychology, will improve patient reported outcomes post testing.

## Summary and considerations for development of predictive testing guidelines for MND

Here, we summarise the literature to highlight the complex issues surrounding predictive genetic testing for MND. Most MND linked genes are associated with autosomal dominant disease; with a 50% chance of offspring inheriting the causal variant. However, penetrance of MND for those with a pathogenic variant varies, being influenced by both genotype and family history. Further, the motor and cognitive phenotype of MND is highly variable, even within a family. The widespread use of next generation sequencing in many healthcare systems has led to the number of people with MND who have a genetic cause identified increasing substantially. Personalised medicine trials targeting presymptomatic individuals with pathogenic variants has focussed attention on this population. Linked to this, there is evidence of an increase in people seeking predictive genetic testing for MND. Despite this, there is no evidence-based guideline for predictive genetic testing in MND and modifications of the HD predictive testing protocol are utilised. Given the complex genomics of MND, we propose that a research-based guideline for predictive genetic testing in unaffected relatives would be valuable. Suggested areas of research required to inform this guideline include greater understanding of penetrance, understanding mechanisms of phenotypic variability, preferred models of genetic counselling among MND families, psycho-social impacts of predictive testing and training needs for clinicians. We recognise that models of care will differ depending on cultural factors and healthcare settings.
